# Adjuvant local antibiotic prophylaxis in Gustilo–Anderson IIIb open fractures: up to 10 years follow-up on clinical outcomes and complications

**DOI:** 10.1186/s13018-025-05765-5

**Published:** 2025-06-23

**Authors:** Eito Asano, Hossai Hemat, Abdullah Bin Sahl, Anand Pillai

**Affiliations:** 1 Trauma and Orthopaedics, University of Manchester, Oxford Road, Manchester, M13 9PL UK; 2 Trauma and Orthopaedics, Wythenshawe Hospital, Manchester University NHS Foundation Trust, Manchester, M23 9LT UK; 3 Royal College of Surgeons in Ireland, 123 St Stephen’s Green, Dublin, D02 YN77 Ireland

**Keywords:** Open fractures, Osteomyelitis, Local antibiotics, Gentamicin, Cerament GHealthcare access, Socio-economic disparities

## Abstract

**Background:**

High-grade open fractures carry a significant risk of osteomyelitis, despite advancements in surgical techniques and treatment protocols. The use of prophylactic, adjuvant local antibiotics is controversially discussed in the literature. CERAMENT®G (BONESUPPORT™) is a novel synthetic bone substitute that effectively elutes gentamicin. This report presents the largest and longest follow-up data collected over the past decade, comparing these findings to our previous findings to identify long-term complications, as well as comparing the outcome against other forms of local antibiotic prophylaxis.

**Methods:**

A retrospective, descriptive analysis was conducted on patients with Gustilo–Anderson IIIb fractures treated with CERAMENT®G as an adjunct to the conventional fix-and-flap approach from June 2013 to August 2021. Patient demographics, orthoplastic interventions, microbiological findings, and the latest outcome data—including union rates, infection rates, mortality, and amputation outcomes—were extracted from electronic records up to April 2024.

**Results:**

Seventy-six patients with 78 fractures were included, with a mean follow-up period of 88.7 months. The primary union was achieved in 65 cases (83.3%), and secondary union following bone grafting was achieved in 5 of 13 non-union cases. Three mortalities were identified. Four cases required amputation due to osteomyelitis, flap failure leading to soft tissue infection, failure in bone reconstruction, and chronic pain. Superficial infections occurred in 33 cases (42.3%), and osteomyelitis in 4 cases (5.1%). No local or systemic adverse reactions, including ototoxicity, were reported.

**Conclusion:**

CERAMENT®G is a safe and effective option for local antibiotic delivery in high-grade open fractures as an adjunct to systemic antibiotic prophylaxis. Its adjunctive use significantly reduced the risk of osteomyelitis compared to systemic prophylaxis alone and was superior to PMMA beads alone.

## Introduction

Osteomyelitis is one of the most concerning complications of high-grade open fractures. Timely administration of systemic antibiotics is critical in preventing deep infections. Despite optimisation in surgical and antimicrobial protocols for managing open fractures over the last decade, the incidence of osteomyelitis and the recurrence rate remains high [1]. A meta-analysis from 2024 involving thirteen RCTs found 13.91 osteomyelitis cases per 100 person-years (95% CI, 8.47–22.84) across all open tibial fractures [2]. Despite systemic therapy, osteomyelitis is reported to have a recurrence rate of 20 to 30% [3]. Physical and mechanical properties of open fractures contribute to suboptimal prevention of deep infections. Periosteal stripping, tissue contaminations, and vascular compromise are key properties [4]. The disruption in the vascular anatomy compromises the ability of systemic antibiotics to reach the target tissue. Therefore, there is a concern that a high serum concentration may be required to maintain local concentrations above minimal inhibitory concentration.

To address this challenge, local delivery of antibiotics in conjunction with systemic therapy has been proposed as a potential solution. Although extensively discussed in the literature, the safety and efficacy of prophylactic local antibiotics remain controversial [5]. Previously described delivery methods include Polymethyl Methacrylate (PMMA) beads [6, 7], local injection of powdered [8] or aqueous antibiotics [9], and antibiotic-coated fixation devices [10]. PMMA beads are one of the pioneering local antibiotics. However, it is unsuitable for fractures with extensive bone void as the contact surface area of the beads limits the elution [11]. Additionally, their non-biodegradable nature necessitates revision surgery for removal, further complicating patient recovery [8].

CERAMENT® G (BONESUPPORT™), a novel synthetic ceramic bone graft substitute, contains 175 mg of gentamicin sulfate per 10 mL of material. In vitro studies have demonstrated its ability to achieve an initial high peak in gentamicin release, followed by a sustained release maintaining concentrations above MIC for over 28 days [12]. This initial high peak of gentamicin release prevents biofilm formation and consequential deep infections [13]. Furthermore, the matrix stimulates a rapid de novo remodeling of bone over 6 to 12 months, aimed at reducing the risks of refractures and non-union.

Over the past decade, the Wythenshawe Hospital Department of Trauma and Orthopaedic Surgery has been monitoring long-term outcomes and complications associated with the use of CERAMENT®G in Gustilo-Anderson (GA) IIIb open fractures managed with a fix-and-flap approach [14–16]. Our previous report on an eight-year follow-up indicated a 3.75% incidence of osteomyelitis, an overall union rate (primary and secondary) of 96%, and a limb salvage rate of 96% [16]. This report builds upon that foundation forming the largest and longest follow up of 3B fractures with the use of adjuvant local antibiotics, supplementing an additional two years of follow-up data while comparing the findings against other methods of local antibiotic delivery to evaluate its efficacy comprehensively. 

## Methods

### Study design and patient selection

This was a retrospective observational study analysing data from patients admitted between June 2013 and August 2021 for the definitive fixation of GA IIIb fractures. All patients underwent a combined fix-and-flap approach supplemented with CERAMENT® G (BONESUPPORT™).

Patients with open fractures amenable to direct wound closure (non-GA IIIB) were excluded from the study. Additionally, cases in which Cerament G was not utilized as the local antibiotic carrier during definitive surgery were not included in the analysis.

### Clinical protocol

The British Orthopaedic Association (BOA)/British Association of Plastic, Reconstructive and Aesthetic Surgeons (BAPRAS) guidelines [17] and the trust’s antimicrobial policy were adhered to in all cases. All patients were understood to have antibiotic administration following the BOA/BAPRAS guidelines for open fractures, which recommend intravenous prophylactic antibiotics ideally within one hour of injury and continued until definitive wound closure, in our institution [17]. All patients received IV Co-amoxiclav before the first debridement until soft tissue closure. For patients with penicillin allergies, a single dose of IV Teicoplanin and Metronidazole was followed by IV Clindamycin and oral Ciprofloxacin. In addition, all patients were administered a weight-adjusted loading dose of IV Gentamicin before debridement. Deep tissue samples were collected for culture and sensitivity during the debridement. Immediately after debridement, temporary external fixators were placed to achieve early bone stabilisation until definitive fixation. Cerament G was then administered at the time of definitive fixation as part of a single-stage fix-and-flap approach, ensuring localized antibiotic delivery during the final reconstructive procedure. To fill the bone void, 10 mL of CERAMENT® G was injected. For cases where immediate soft tissue coverage was not possible, vacuum-assisted closure (VAC) dressings were utilised until definitive soft tissue closure was achieved.

### Follow-up protocol

Patients were monitored in regular outpatient clinic visits to assess the progress of bony union. Radiological assessments were performed at three, six, and twelve months post-operation to confirm union.

### Data collection and outcomes

Patient demographics, comorbidities, orthoplastic interventions, microbiological findings, and clinical outcomes were extracted from electronic patient records in December 2024. Long-term follow-up was performed by reviewing clinic letters generated or transferred from external centres to date. All patient identifiers were anonymised, and previous records were cross-checked for errors or duplicating entries.

Primary outcomes are the incidence of infectious complications and long-term adverse reactions. Secondary outcomes encompass rates of primary union, requirements for additional bone-supporting procedures, non-union rates, secondary union, and incidences of amputation.

### Data analysis

Patients with polytrauma involving fractures at separate anatomical sites (e.g. femur and tibia) were recorded as separate fractures, while concurrent tibial and fibular fractures were treated as a single fracture. The collected data were compared against systemic antibiotic prophylaxis alone and other local antibiotic elution devices within the confines of descriptive analysis.

## Results

One-hundred and twenty-seven (127) patients were admitted between June 2013 and August 2021 for definitive management of GA IIIb open fractures. Seventy-Eight (78) were treated using Cerament G. Two patients were excluded from the analysis as CERAMENT G was used to fill the bone void in non-union rather than primary fracture management. Reviewing the previously collected data, one pair of duplicating entries was removed.

In total, 76 patients and 78 fractures were included in the final analysis. The median age of the cohort was 35.3 years (range: 9.3–88.7 years; interquartile range [IQR]: 27.3 years). The majority of the patients were male (*n* = 62; 79.5%). Nineteen patients were classified as overweight (BMI 25.0–30.0 kg/m^2^), while 20 patients were obese (BMI > 30.0 kg/m^2^) (Table 1). Nearly half of the cohort were current smokers (46.2%).

**Table 1:** Patient demographics

Patient demographics (*N* = 78)			
Age [median yr (range, IQR)]		35.3	(9.3–88.7, 27.3)
Male Sex		62	(79.5%)
BMI			
Ideal	18.5–24.9	26	(33.3%)
Overweight	25–30	19	(24.4%)
Obese	> 30	20	(25.6%)
No BMI Available		9	(11.5%)
Paediatric Patients		4	(5.1%)
Current Smoker		36	(46.2%)
Charlson Comorbidity Index			
0–1		66	(84.6%)
2–3		6	(7.7%)
> 4		6	(7.7%)

Most fractures scored less than one on the Carlson Comorbidity Index (84.6%), representing an estimated 10-year survival probability of greater than 96% [18]. Among notable comorbidities, six patients had diabetes mellitus, and three were current intravenous drug users. Only one case involved a patient who was immunosuppressed at the time of injury. This patient had a history of long-term treatment with prednisolone, ciclosporin, and mycophenolate.

No patients reported known allergies to aminoglycosides. Five patients (6.4%) had documented penicillin allergies, and one patient had a known tetracycline allergy.

Thirty-one cases were discharged prior to the 12-month follow-up. Eight cases were followed up in external hospitals, Nine were discharged following confirmation of early union, One following uncomplicated amputation, Two due to death, and 11 due to loss to follow-up. Of those thirty-one cases, nine had further radiology follow-up beyond 12 months to assess bony healing but were not formally seen in the clinics.

### Injury and treatment

Road traffic collisions were the most common mechanism of injury, accounting for 43 cases (55.1%), followed by falls from height (refer to Appendix). Segmental bone defects were present in 42 fractures (53.8%), while 36 fractures exhibited contained bone defects. The majority of fractures occurred in the tibia and fibula (73.1%) (Table 2).

**Table 2:** Fracture characteristics

Site	Intervention	N	(%)
Tibia and Fibula	IM Nail	14	(17.9)
	ORIF	21	(26.9)
	ExFix	22	(28.2)
	Total	57	(73.1)
Femur	ORIF	3	(3.8)
Ankle	IM Nail	1	(1.3)
	ORIF	5	(6.4)
	ExFix	4	(5.1)
	Total	10	(12.8)
Foot	IM Nail	1	(1.3)
	ORIF	2	(2.6)
	ExFix	1	(1.3)
	PP	3	(3.8)
	Total	7	(9.0)
Radius	ORIF	1	(1.3)

*IM Nail * Intramedullary Nail, *ORIF* Open Reduction and Internal Fixation, *ExFix* External Fixators, *PP* Percutaneous Pins

The median time from injury to primary debridement was 10 h (range: 3–40 h; IQR: 8 h). Forty-eight cases (61.5%) underwent debridement within the first 12 h after admission. Nearly all injuries (96.2%) were debrided within 24 h, with the exceptions being two fractures delayed due to theatre unavailability and one case where the patient experienced cardiac arrest while awaiting transfer to theatre.

Definitive fixation was achieved at a median of eight days post-fracture (range: 1–53 days; IQR: 5 days). Three cases experienced prolonged delays of 35, 37, and 53 days due to the injuries being sustained overseas. In these instances, initial debridement was performed in the respective countries of injury, and definitive fixation was deferred until the patients returned to the UK.

Soft tissue coverage techniques were tailored to each case, based on the extent of soft tissue loss, injury site, and graft availability. The most frequently employed method was the free anterolateral thigh flap (43.6%), followed by the fasciocutaneous local flap (30.8%). Additional methods included skin grafts, gastrocnemius local flaps, gracilis flaps, and latissimus dorsi flaps. Four cases did not require flap or skin graft coverage.

The median duration of radiological follow-up was 26.5 months (range: 1.5–101 months; IQR: 52.75 months). Clinical follow-up had a median duration of 18.5 months (range: 0.8–107 months; IQR: 32.75 months). The mean overall study follow-up period, defined as the time between surgery and data collection, was 88.74 months.

### Bone void filling and union

Fracture healing was assessed using both functional clinical evaluations and radiological imaging (X-ray/CT). Bone void filling was monitored through serial imaging at the 3rd, 6th, and 12th months post-treatment.

Primary union was achieved in 65 fractures (83.3%). Among these, 40 fractures achieved union without the need for additional interventions or stimulation. Delayed union was observed in 24 cases (30.8%), and union was eventually confirmed in all but two of these fractures. Of the two exceptions, one patient was lost to follow-up, while the other exhibited slow fracture healing complicated by flap failure, leading to recurrent soft tissue infection. This patient was advised to undergo amputation but unfortunately died before the procedure could be performed.

Supportive interventions were required in seven patients with delayed union. These included EXOGEN® (Bioventus), bone grafting, and LIPOGEMS® (Norcross), all of which contributed to successful fracture healing.

Non-union was observed in 13 fractures (16.7%). One additional case was suspected to have non-union but was lost to follow-up after achieving only 10% bone void filling by the 6th month. Among the 13 confirmed non-union cases, secondary union was achieved in five (38.5%) following further intervention. Three of these patients underwent bone grafting, all achieving secondary union by the 12th month.

One patient with a distal femoral fracture underwent total knee replacement after non-union was confirmed at six months. However, this patient subsequently sustained a peri-prosthetic refracture after 12 months.

### Limb salvage

Amputation was performed in three patients (3.8%) due to flap failure leading to osteomyelitis, flap failure resulting in soft tissue infection, and failure in bone reconstruction. The amputations were carried out at 16, 45, and 20 days following definitive fixation, respectively.

One patient with chronic osteomyelitis was scheduled for amputation during a six-month follow-up; however, this patient missed their pre-operative assessment and subsequent appointments and passed away 14 months after definitive fixation. Additionally, one patient underwent an elective amputation three years after definitive fixation due to chronic pain.

### Mortality

There were three mortalities recorded within the twelve-month follow-up period. The cause of death was identified in two patients and was not directly related to the primary injury or orthoplastic intervention. One patient succumbed to coagulopathy secondary to acute liver failure, in the context of a history of hepatitis C infection, and another due to a cerebrovascular accident. The third patient, who achieved early union and functional recovery, was discharged three months post-surgery but later died following discharge.

The Charlson Comorbidity Index (CCI) scores for the deceased patients were three, four, and six, corresponding to estimated ten-year survival rates of 77, 53, and 2%, respectively [calculated as 10-year survival = 0.983^(eCCI × 0.9)]. [18]. While one mortality case involved concurrent flap failure and soft tissue infection, none of the deaths were associated with metalwork infection or osteomyelitis.

### Adverse drug reactions and long-term complications

No adverse drug reactions to gentamicin were reported, in particular, no reported cases of ototoxicity.

Chronic pain necessitated elective amputation in three cases. One case returned to the clinic three years later with ongoing discussion for amputation. One case returned due to chronic pain six years post-injury, with nerve conduction study revealing sciatic neuropathy with sensory and motor axonal loss.

Three cases of limb shortening were reported, with two successfully treated through limb lengthening procedures. Additionally, two cases of refractures occurred after the union was achieved.

One patient returned with a recurrent leg ulcer due to underlying peripheral vascular disease. However, both wound swab and imaging studies did not suggest an infected ulcer or osteomyelitis.

Osteoarthritic changes were seen in six cases. One patient who sustained a Pilon fracture returned six years following the injury due to chronic pain secondary to osteoarthritis at the tibiotalar joint.

As in the Cerament G BONESUPPORT™ instructions manual, early wound oozing and white wound discharge is a recognised potential complication of calcium sulfate bone void fillers in the first few days [19]. Inflammatory reactions have also been documented with their use. Despite these potential concerns and the limitations of our retrospective data colleciton, no significant complications related to wound healing and discharge were observed in our patient population.

### Microbiology, soft tissue infections, and osteomyelitis

Deep tissue samples for culture and sensitivity were collected before definitive fixation in all patients. Most fractures (*n* = 69, 77.2%) showed no microbial growth after 48 h of incubation. Coagulase-negative *Staphylococcus* (CONS) was isolated in four fractures, and six cases exhibited mixed microbial growth.

Gentamicin resistance was observed in only one fracture, which showed a mixed growth of *Aeromonas salmonicida*, *Enterococci*, *Citrobacter freundii*, and *Shewanella* species. This fracture developed chronic osteomyelitis, confirmed via MRI seven years later. Further details of this case were unavailable, as the patient’s care was transferred to another centre.

- Infective complications were categorised into superficial infections and osteomyelitis. Superficial infections included soft tissue and pin-site infections, while osteomyelitis was diagnosed based on clinical and radiological evidence supported by bone tissue sample analysis.
- Superficial Infections: There were 33 cases of superficial infections (42.3%), comprising 30 wound or flap infections and three pin-site infections. Most infections (*n* = 31, 93.9%) were successfully treated with oral antibiotics, with or without additional surgical interventions, including debridement, flap revision, and metalwork removal as needed. The most common causative organism was methicillin-sensitive Staphylococcus aureus (MSSA) in 14 cases, followed by Enterococcus cloacae in three cases. However, the specific oral antibiotic agents used were not recorded.
- Osteomyelitis: Four cases of osteomyelitis (5.1%) were identified. Two cases showed positive deep tissue cultures during initial debridement: one grew CONS, and the other was polymicrobial, involving *Aeromonas salmonicida*, *Morganella morganii*, *Shewanella* spp., and Enterococcus spp. One patient with osteomyelitis had underlying immunosuppression at the time of injury, and another was associated with gentamicin-resistant CONS (*Staphylococcus epidermidis*). One case required a below-knee amputation due to osteomyelitis. The remaining uncomplicated osteomyelitis cases were resolved successfully, with the eradication of infection and confirmed union by twelve months (Table 3).

**Table 3:** Summary of patient characteristics in fractures which developed osteomyelitis

	Fracture #60	Fracture #67	Fracture #92	Fracture #96
BMI	22.2	22.6	26.3	43.6
Smoking	Non-Smoker	Current Smoker	Non-Smoker	Ex-Smoker
CCI	1	0	3	3
Comorbidity	Psoriasis	Epilepsy	T2DM	HTN, T2DM
Site	Proximal Tibia	Distal Tibia	Mid Shaft Tibia	Ankle
Aetiology	Assault with hammer	Fall from bed	Fall from Farming Tractor	No data
Bone Defect	Segmental	Segmental	Segmental	Contained
Time to Debride (hr)	10	3	5	6
Time to Fixation (d)	11	7	5	1
Fixation	ORIF	IM Nail	Ex-Fix	ORIF
Flap	Gastrocnemius Local Flap	Fasciocutaneous Local Flap	Skin Graft	Fasciocutaneous Local Flap
Microbiology before fixation	No growth	No growth	Mixed Growth	No growth
Gentamicin Sensitivity	n/a	n/a	Resistant	n/a
Time to OM	16 months	4 months	7 years	12 months
OM Organism	No data	MSSA	No data	Pseudomonas
Outcome	Eradication of OM / Union	Eradication of OM / Union	Amputation	Eradication of OM / Union / Severe Residual Osteoarthritis

## Discussion

This study provides a comprehensive analysis of the long-term outcomes and complications associated with the use of the novel local antibiotic-elution device, Cerament G, in managing GA IIIb open fractures using a fix-and-flap approach. Key outcomes, including rates of primary and secondary union, amputation, mortality, and infectious complications, were examined over a twelve-month period and beyond.

Table 4 provides a summary comparing our current data with our previous findings. Additionally, a selection of prior studies was reviewed to evaluate the outcomes associated with alternative methods of local antibiotic delivery. While no formal selection criteria were applied, the reviewed literature was chosen based on the use of aminoglycosides as the primary antibiotic agent.

**Table 4:** Summary of our result and comparison to existing literature

Study	GA	N	Treatment	Mean Study Follow-Up (m)	Superficial Infection	Osteomyelitis	Primary Union	Amputation
Gopal (2000)	III b, c	84	Systemic Only	-	6.0% (5/84)	9.5% (8/84)	60.7% (51/84)	4.8% (4/84)
Lawing (2015)	I, II, III	151	Systemic + Local Injection ^a^	11.3	4.0% (6/151)	6.6% (10/151)	85.7% (144/168)	Excluded
		183	Systemic Only	12.5	5.5% (10/183)	14.2% (26/183)	84.7% (155/183)	Excluded
Moehring (2000)	III a, b, c	24	Tobramycin Impregnated PMMA Beads Only	15	-	8.3% (2/24)	87.5% (21/24)	0
Perisano (2021)	III a, b, c	17	Systemic + Abx coated nail ^b^	24.1	11.8% (2/17)	11.8% (2/17)	88.2% (15/17)	0
Jahangir (2019) ^†^	III b	52	Systemic + Cerament G	13.9	-	0 ^‡^	84.3% (43/51)	2.0% (1/51)
Aljawadi (2020) ^†^	III b	80	Systemic + Cerament G	22	20.0% (16/80)	2.5% (2/80) ^‡^	88.3% (68/80)	3.8% (3/81)
Henry (2023) ^†^	III b	81	Systemic + Cerament G	55	44% (36/81)	3.7% (3/80) ^‡^	82% (64/80)	3.8% (3/80)
Our Result	III b	79	Systemic + Cerament G	88.7	42.3% (33/78)	5.1% (4/78)	83.3% (65/78)	3.8% (3/78)

^a^Aqueous Solution of Gentamicin (40 mL of 2 mg/mL) ± Further irrigation with 0.5 mg/mL mixture of normal saline and gentamicin

^b^EXPERT ™ Tibial Nail PROtect ™, DePuy Synthes (Gentamicin Coated Tibial Nail)

^†^Our previous reports

^‡^Note discrepancy in our study size is due to an exclusion of two patients from our previous study, a deletion of duplicated entry, and a separation of one case to two distally sited fractures

### Follow-up from previous data

Several changes to the study cohort are worth noting. Two patients were excluded because Cerament G was used as a void filler for non-union fractures rather than as a primary prophylactic measure. Additionally, one patient died prior to the three-month follow-up, and no data on fracture healing or complications were available for inclusion. A duplicative entry was removed, and one patient with polytrauma (femur and ankle fractures) had their cases analysed as two separate fractures.

After accounting for the adjusted sample size, there were no observed changes in the rates of superficial infection, primary union, or amputation compared to the previous data. However, one additional case of osteomyelitis and one new amputation were identified. Notably, no systemic reactions or gentamicin-related toxicities were reported in this updated cohort.

### Comparison to other forms of local antibiotics

The incidence of osteomyelitis in this study was comparable to other methods of local antibiotic delivery, including aqueous gentamicin injections [20], polymethylmethacrylate (PMMA) beads [6], and antibiotic-coated intramedullary nails [10]. These findings align with prior in vitro and clinical evidence indicating that Cerament G achieves levels of gentamicin elution equivalent to antibiotic-loaded beads [12]. Importantly, all reviewed studies consistently demonstrated that local antibiotic delivery is superior to systemic antibiotic therapy alone in preventing osteomyelitis.

However, the incidence of soft tissue infections was higher in this study compared to those using alternative methods. This discrepancy may be attributed to differences in inclusion criteria. Unlike other studies that excluded patients with established deep-seated infections at the time of initial debridement, this study also included all patients with positive deep tissue cultures from the first debridement. While this may explain the higher infection rates, other factors can also be considered. It is possible that Cerament G may be less effective at preventing soft tissue infections compared to other local antibiotic adjuvants. Additionally, calcium sulfate-based bone void fillers have been associated with local tissue inflammation and wound drainage, which may contribute to increased soft tissue infection rates. A prospective cohort analysis is warranted to determine whether the inclusion of patients with primary infections has biased the observed rates of soft tissue infection and to further evaluate the role of these alternative contributing factors.

The primary union rate of 83.3% in this study was consistent with rates reported in studies investigating alternative local antibiotic methods for GA III fractures. Beyond its antibiotic-elution properties, Cerament G contains hydroxyapatite, a known osteoconductive molecule included to support bone remodeling. However, our data do not provide direct evidence that the hydroxyapatite component specifically contributed to bone healing in this cohort. Although this study included fractures with varying extents of bone voids, data on initial bone void size were not consistently available in the comparative studies, limiting direct comparisons of bony healing and remodeling rates.

Limb salvage rates were challenging to compare directly due to differing study designs. Some studies, especially those requiring long-term follow-up, excluded patients who underwent amputation. This study’s limb salvage rate was comparable to that reported by Gopal et al. [21]. The absence of amputations in the smaller cohort studied by Perisano et al. [10] may reflect either a genuine superiority of antibiotic-coated intramedullary nails in preventing limb-threatening complications or differences arising from small sample sizes.

### Limitations

The retrospective nature of this study introduces certain limitations. Wythenshawe Hospital serves as the tertiary trauma center for South Manchester, and some patients were followed up at their local district general hospitals or by external orthopedic teams. Minor complications managed at external centers may not have been captured, introducing the potential for under-reporting complication rates. Loss to follow-up was another limitation, as some patients missed clinical and radiological follow-up appointments. Among four patients who missed the twelve-month follow-up, only one demonstrated satisfactory bone void filling by the nine-month mark. This further exacerbates the risk of under-reported complications. However, the risks above were mitigated through the use of digital platforms such as EPIC and Sectra PACS, which allow cross-site visibility of patient records and regional radiological data to ensure comprehensive data collection.

Selection bias and unmeasured confounders, such as socioeconomic status, therapy engagement, and proximity to the hospital, may also influence outcomes in this single-center study. Although all patients receiving Cerament G for GA IIIb fractures were included, this dataset may not fully capture broader demographic or clinical variations.

Additionally, variability in study designs and management protocols limits the comparability of this study’s findings to those in the literature. Differences in technical details, such as irrigation methods and soft tissue coverage, as well as inclusion criteria, pose confounders. For instance, Lawing et al. [20] excluded patients who underwent amputation, while this study included such cases. The decision to amputate in the context of open fractures depends on factors such as limb-threatening infections or non-union, which are difficult to standardise across studies. Conducting a comprehensive meta-analysis of local antibiotic therapies falls beyond the scope of this original research. The comparative studies included in this work were utilised solely to provide a preliminary benchmark of performance.

Time-to-antibiotic administration is a well-established prognostic factor in infection prevention after open fractures [22]. While intravenous prophylactic antibiotics were administered in accordance with BOA/BAPRAS guidelines [17], which recommend initiation within one hour of injury and continuation until definitive wound closure, a noted limitation is the absence of data to confirm this adherence at individual patient level. Such data review was not part of our retrospective record analysis. Given that hospital and emergency protocols are designed to follow these guidelines, it was assumed that all patients received antibiotics as per BOA/BAPRAS guidelines.

As previously mentioned, early wound leakage is a recognised complication associated with calcium sulfate-containing bone void fillers. The association of this with wound pathology was difficult to assess due to the limitation of our retrospective data analysis. It was not possible to assess this from the data collection as the complication is short-term from the product in the first few days. Despite this, no cases in our study exhibited significant impairments in wound healing to demonstrate a potential link to wound leakage.

A key strength of this study is the inclusion of 10-year data, which is particularly valuable in this patient population where long-term follow-up can be challenging. However, a limitation is the lack of a comparative arm, which limits the ability to directly assess the intervention’s effectiveness against other treatment modalities. Instead, comparisons are drawn indirectly from existing literature. While this provides some context, it does not substitute for a controlled comparison. Future research should incorporate comparative studies to better establish the relative efficacy of this approach.

### Future directions

Prophylactic local antibiotic use remains an evolving area in the management of open fractures. While this study provides valuable insights into the long-term outcomes associated with Cerament G, further research is needed to determine whether specific local antibiotic delivery systems confer superior outcomes. Large-scale, prospective cohort studies with a comparative arm or robust meta-analyses are necessary to address the limitations inherent in retrospective designs and to establish evidence-based best practices for using local antibiotics in open fracture management.

## Conclusion

This study updates our previously published findings on the use of CERAMENT®G (BONESUPPORT™) in managing Gustilo-Anderson IIIb fractures through a fix-and-flap approach, demonstrating its efficacy in preventing osteomyelitis and supporting bone healing. With a primary union rate of 83.3%, a limb salvage rate of 96.2%, and an osteomyelitis incidence of 5.1%, CERAMENT® G showed comparable or superior performance to other local antibiotic delivery systems while also providing osteoconductive benefits. Although the soft tissue infection rate was 42.3%, most cases were resolved with antibiotics and surgical interventions. These results underscore the potential of CERAMENT® G as a reliable adjunct for high-grade open fractures, with further prospective, multi-center studies needed to confirm its long-term safety and efficacy.

## Data Availability

No datasets were generated or analysed during the current study.

## Appendix

See Tables

**Table 5:** Aetiology

Aetiology	N	(%)
Road traffic collision	43	(55.1%)
Bike vs Car	6	(7.7%)
Crush Injury	3	(3.8%)
Fell from moving vehicle	3	(3.8%)
Pedestrian vs Car	2	(2.6%)
Pedestrian vs Train	1	(1.3%)
Unspecified	28	(35.9%)
Fall from height	18	(23.1%)
Other	6	(7.7%)
Blast Injury	2	(2.6%)
Assault	1	(1.3%)
Concrete cutter	1	(1.3%)
Gunshot	1	(1.3%)
Sport injury	1	(1.3%)
No data available	11	(14.1%)

5,

**Table 6:** Soft tissue coverage

Soft Tissue Coverage	N	(%)
Free Anterolateral Thigh Flap	34	(43.6)
Fasciocutaneous local flap	24	(30.8)
Skin Graft	7	(9.0)
No flap	4	(5.1)
Gastrocnemius Local Flap	4	(5.1)
Gracillis Muscle Flap	3	(3.8)
Latissimus Dorsi	2	(2.6)

6 and

**Table 7:** Microbiology of deep tissue samples before definitive fixation

Microbiology from Deep Tissue Sample	N (%)
No growth	61 (77.2%)
Mixed Growth	6 (7.6%)
CONS	4 (5.1%)
MSSA	2 (2.5%)
Enterobacter cloacae	2 (2.5%)
Others	
MRSA	1 (1.3%)
Proteus mirabilis	1 (1.3%)
Enterobacter amnigenus	1 (1.3%)
Stenotrophomonas maltophilia	1 (1.3%)

CONS = Coagulase-negative Staphylococci; MSSA = Methicillin sensitive Staphylococcus aureus; MRSA = Methicillin resistant Staphylococcus aureus

7.
