# Correction: Adjuvant local antibiotic prophylaxis in Gustilo–Anderson IIIb open fractures: up to 10 years follow-up on clinical outcomes and complications

**DOI:** 10.1186/s13018-025-06104-4

**Published:** 2025-07-25

**Authors:** Eito Asano, Hossai Hemat, Abdullah Bin Sahl, Anand Pillai

**Affiliations:** 1 Trauma and Orthopaedics, University of Manchester, Oxford Road, Manchester, M13 9PL UK; 2 Trauma and Orthopaedics, Wythenshawe Hospital, Manchester University NHS Foundation Trust, Manchester, M23 9LT UK; 3 Royal College of Surgeons in Ireland, 123 St Stephen’s Green, Dublin, D02 YN77 Ireland

**Correction to: Journal of Orthopaedic Surgery and Research (2025) 20:61**0

10.1186/s13018-025-05765-5

In this article [1], the heading “Comparison to other forms of local antibiotics” under the section “Discussion”, the text “It is possible that Cerament G is less effective at preventing deep infections compared to other local antibiotic adjuvants.” should read as “It is possible that Cerament G may be less effective at preventing soft tissue infections compared to other local antibiotic adjuvants.”.

The original article has been corrected.

